# Platelet-rich plasma therapy: key infection prevention practices and strategies for safety risk reduction

**DOI:** 10.1017/ice.2025.10316

**Published:** 2026-01

**Authors:** Rebecca A. Stern, Jennifer Andrews, Katherine Bashaw, Thomas R. Talbot

**Affiliations:** 1 Division of Infectious Diseases, Department of Medicine, Vanderbilt University Medical Center, Nashville, TN, USA; 2 Department of Pathology, Microbiology & Immunology, Vanderbilt University Medical Center, Nashville, TN, USA; 3 Department of Pediatrics, Vanderbilt University Medical Center, Nashville, TN, USA; 4 Department of Infection Prevention, Vanderbilt University Medical Center, Nashville, TN, USA

## Abstract

**Background:**

Platelet-rich plasma (PRP) injections are increasingly performed in outpatient settings to treat select musculoskeletal injuries, arthritis, hair loss, and wounds. There is a need for procedure-specific guidance and standardization of PRP practices to mitigate associated infection prevention (IP) risks such as bloodborne pathogen exposure and unsafe injection use.

**Objective:**

Develop a standardized approach for PRP administration which incorporates existing IP regulatory and professional society guidance.

**Methods:**

Observation and descriptive review of PRP injection protocols across subspecialties at a tertiary medical center, focused on ambulatory IP and regulatory standards compliance. Development of a standardized operating procedure (SOP) to mitigate IP risks and align with regulatory guidance.

**Results:**

Observations were completed in orthopedic, wound care, and oral maxillofacial surgery clinics. Variability in practice was noted for product labeling, centrifugation, and injection modalities. A multidisciplinary workgroup convened to develop and operationalize an SOP. Classification of PRP as a blood product introduced nuances to protocols for product preparation, handling, administration, labeling, and documentation to comply with regulatory standards.

**Conclusions:**

Development and implementation of an SOP for PRP treatment requires an awareness of the scope of practice in a healthcare system and identification of pertinent regulatory standards for integration into workflows. Partnerships between IP teams, subspecialty clinical providers, blood safety experts, quality and safety teams, and healthcare technology are essential to minimize variability in practice, ensure safety of patients and healthcare personnel, and align with regulatory standards.

## Manuscript

## Background

Platelet-rich plasma (PRP) treatments are increasingly performed in outpatient settings to promote wound healing and regeneration of tissues and bones. Wide subspecialty use is encountered in orthopedics, dermatology, wound care, plastic surgery, and dentistry. Prepared from autologous blood by phlebotomy, PRP is then centrifuged to a final product containing high platelet concentrations which are thought to release beneficial growth factors.^1,2^ Approaches to PRP therapy technique, dosing concentrations, formulation, and optimal delivery format are highly variable in the literature.^3,4^ Absence of a standardized protocol by professional societies and its broad spectrum of use creates challenges to systematically align practices, both across and within subspecialties, with regulatory standards, infection prevention (IP), and safety precautions.

Existing data and guidance by professional societies on PRP use are limited regarding potential infection control and safety risks. The nuances of managing a blood product are prudent to incorporate into risk evaluation and logistics, noting that PRP is classified as a blood product per the Association for the Advancement of Blood & Biotherapies (AABB).^5^ Risks are amplified by the wide scope of practice, lack of consensus on preparation, variable supplies, inconsistent training of healthcare personnel (HCP), and lack of national standardized protocols.

This review explores the authors’ own institutional experience investigating the scope and variability in PRP practices, as well as developing guidance for best practices pertaining to IP and safety with reference to existing regulatory requirements, literature, and feasibility of implementation. Collaboration with key stakeholder groups of subspecialists, quality and safety teams, blood safety experts, and healthcare technology is emphasized to generate consensus and operationalize best practices.

## Methods

PRP came to the attention of the institutional IP team during an environment of care survey in an orthopedic clinic. Gap analysis revealed opportunities to thoroughly assess the scope of PRP practices across the tertiary health system (which has an academic affiliation and consists of over 400 ambulatory clinics), evaluate existing protocols, identify potential infection and safety risks (e.g., hand hygiene, mitigating bloodborne pathogen (BBP) exposure, personal protective equipment [PPE]), align with regulatory requirements, and address clinic infrastructure to support safe administration of PRP.

### Assessment of the scope of PRP practices

First, to understand the breadth and target locations where PRP was occurring across an academic medical system, current procedural terminology (CPT) codes for the prior 12 months (September 2023–2024) were queried per department. Specific CPT codes included several from the Centers for Medicare and Medicaid Services for PRP injection and wound care (Table 1). The overwhelming majority of cases captured (approximately 500) were performed in orthopedic sports medicine clinics, which became the primary focus of the project. Approximately 25 observations by IP team members including infection preventionists, the ambulatory healthcare epidemiologist, and ambulatory quality improvement leaders (associate nursing officer for orthopedic clinics, healthcare informatics specialist) were performed in the main orthopedic sports medicine clinic from December 2024 to April 2025, with additional observations completed in wound and oral/maxillofacial surgery clinics.

**Table 1.** Current procedural terminology (CPT) code examples to report PRP

Clinical category	Code
Orthopedics	0232T^*^
Physical medicine and rehabilitation	
Chronic wound/ulcer care	G0460 (non-diabetic), G0465 (diabetic)
Dental	D7921^**^
Each unit of PRP	P9020

Codes defined by Centers for Medicare and Medicaid Services.^29^ List may not be all inclusive.

* 0232T includes injection(s) at any site, including harvesting and preparation

** Dental code D7921 is distinct from a medical CPT code (e.g., 0232T) and may affect insurance reimbursement.

### Investigation of safety risks and regulatory standards

The IP team investigated PRP risks, safety, and regulatory readiness at sites that performed PRP. PRP process mapping involved direct observations of procedural steps (including set up and post-procedure), procedure kit components (e.g., needles, syringes for phlebotomy and aspiration following centrifugation, separator and concentrator devices), clinical environments, centrifugation, and understanding which HCP performed each step. Multidisciplinary stakeholders collaborated on proposed best practices to develop a standardized operating procedure (SOP) to establish minimum requirements for ensuring patient and HCP safety, align with available recommendations by professional societies and manufacturer’s instructions for use (IFU).

## Results

**Figure 1.**
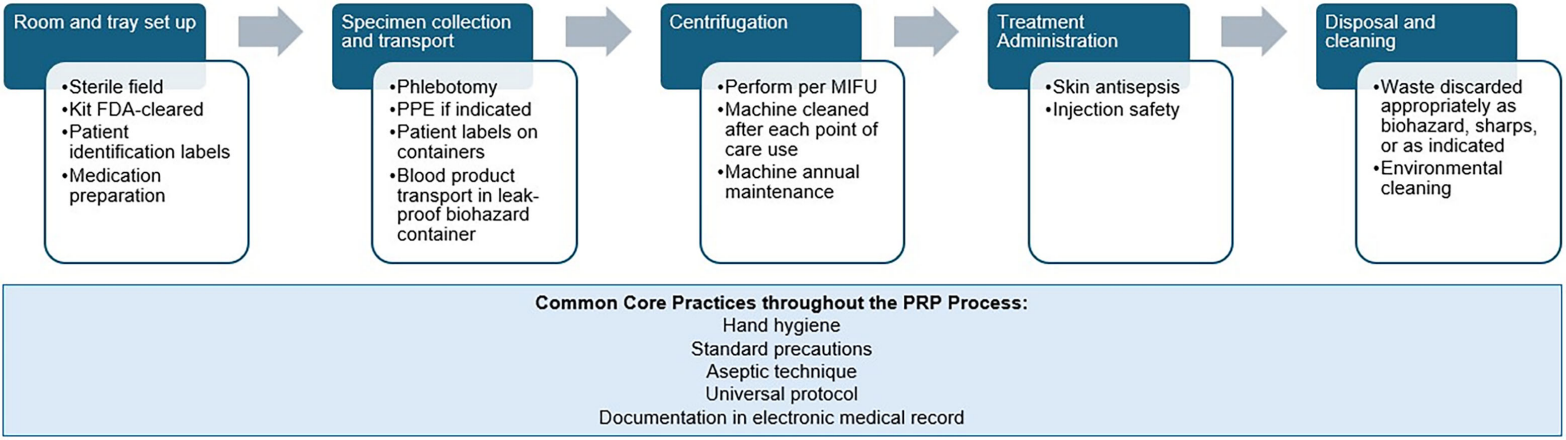
Process flow and key expected steps in the PRP process. Abbreviations: FDA, Food and Drug Administration; PPE, personal protective equipment; MIFU, Manufacturer’s Instructions for Use.

An internal comprehensive audit of PRP practices (Figure 1 and Supplement Table 1 audit tool) identified potential risks and informed recommendations for standardization. Core strategies to address potential issue categories uncovered during the audit are described below. A small subset of SOP guidance is only applicable to certain PRP cases, such as topical (rather than injection) application for wound therapy.

### Bloodborne pathogen (BBP) transmission

The PRP process involves several steps that may increase the risk for BBP exposure, namely phlebotomy, product preparation via centrifugation and treatment injection. Unique to PRP, the blood specimen collected from the patient must be centrifuged at the point of care to concentrate platelets to the desired degree, then injected, typically into joints or tissues. Potential issues of centrifuge cleaning and protocols to handle specimens if containers were disrupted inside the device were identified.

The PRP improvement team developed the following recommendations for PRP practice focused on prevention of BBP transmission:

- Gloves are worn during phlebotomy, blood product handling, centrifugation, and PRP administration whether by injection or topical route.^6^
- Single use kit components and centrifuge tubes are disposed into biohazard containers.
- If ultrasound guidance is used for phlebotomy or percutaneous injection, a sterile probe cover is recommended to mitigate risk of BBP contamination. There is not a firm mandate on ultrasound high-level disinfection (HLD) practices by professional societies for this niche application. AABB defers to each organization (i.e., hospital or healthcare group) to define its equipment cleaning practices.^7^ Practically, this may be difficult to implement depending on availability of ultrasound probes (potential added cost), compatibility of ultrasound probes with HLD chemical agents (alcohol may damage or degrade probe materials, further increasing the risks of adverse event or infection), and IFU requirements.
- Centrifuge processes
  - The centrifuge is ideally situated in the same room where the patient will undergo PRP. If the centrifuge is in a room separate from the patient, the PRP product should be transported in a labeled container to prevent leakage, such as a biohazard bag (referencing Occupational Safety and Health Administration [OSHA] guidance on blood specimens or those containing potentially infectious materials).
  - Centrifuge cleaning and preventive maintenance are performed per the manufacturer’s IFU guidance unique to each model and should be promptly performed for any visible blood or specimen spillage or contamination of the instrument.
- If additional tissue or bone components are utilized, such as platelet-rich fibrin for dental socket preservation or adjunctive bone grafts, the provider should adhere to institution and Food and Drug Administration (FDA)-specific guidance of those samples including obtaining, transporting, storing, handling, disposing, high-level disinfection, and sterilization if indicated.

### Medication safety

Recommendations to mitigate risks of medication safety issues, such as unsafe injection practices, labeling of kit components and the PRP product, documentation in the electronic medical record (EMR), and timing of medication preparation, were developed by the PRP improvement team:

- Injection safety practices outlined by CDC which are relevant to PRP include^8^
  - Aseptic technique when preparing and administering injections (medications and PRP product).
  - Favor the use of single-dose vials. If multi-dose vials are used in a patient treatment area they should be discarded after each patient.
- Labeling and documentation in the EMR
  - Required for medication use, kit components (and expiration date), phlebotomy, and PRP product following centrifugation. Pertinent professional society requirements include:
    - The Joint Commission National Patient Safety Goals for using two patient identifiers.^9^
    - AABB and FDA requirements to capture kit lot numbers, expiration dates, HCP who collected and administered PRP components.^7^
  - Stakeholder feedback identified this as the primary perceived barrier to workflow, underscoring the value of collaborative implementation with healthcare technology teams.
- If anticoagulation (added to the syringe prior to phlebotomy) and/or local anesthetic (applied to the site of injection prior to PRP treatment) are utilized, preparation should occur within a specified time frame prior to use and in accordance with medication safety per the institution’s internal policies. This is intended to avoid delayed time between medication preparation and administration that risks contamination with microorganisms or environmental elements.

### Hand hygiene, standard precautions, and environment of care

Hand hygiene is required prior to each step of PRP, as with other patient care procedures.^10^ Additional guidance by the PRP improvement committee to ensure proper precautions includes:

- Handwashing stations and a sink are required to be present in the room where PRP will occur per the Facilities Guidelines Institute (designation 2.1–3.8.7).^11^
- While performing phlebotomy or any PRP step, if the provider anticipates splashes or sprays, PPE is recommended by CDC to prevent blood or body fluids from contacting the face, mucous membranes, or skin.^12^
- Aseptic technique for joint injections includes skin antisepsis, such as with chlorhexidine or iodine.
- Sterile set up should occur as close to the procedure time as feasible. If done in advance, a sterile drape should cover the field to prevent contamination.

### Regulatory standards and guidelines

Relevant references to standards by professional and regulatory agencies, where available, were paired with recommendations as supporting evidence. Importantly, regulatory oversight of PRP hinges on the fact that it is treated as a blood product per AABB. As a blood product, PRP regulatory oversight falls under the United States FDA Center for Biologics Evaluation and Research (CBER), though is exempt from the 21 Code of Federal Regulations 1271.15(b) standard as an autologous product that is readministered to the patient during the same procedure.^13,14^

Tracking PRP device use is necessary in the event of a component recall, malfunction, or infectious outbreak, all of which require reporting. Ensuring compliance with FDA labeling requirements for PRP in our healthcare system relied upon expertise of operational teams and drew experience from transfusion teams for integrating similar metrics into EMR workflows.

PRP devices (e.g., kits, preparation systems, centrifuges) require FDA clearance prior to being marketed, typically pursued by the 501(k) statute of the Food, Drug and Cosmetic Act for lower risk devices.^15^ PRP treatment is considered largely investigational at this time and not yet FDA approved, meaning that clinical use is at the discretion of the provider if deemed in the best interest of the patient. The distinction between clearance versus approval with respect to regulatory requirements and clinical application introduces complexity, as PRP treatment may be considered “off-label” if used in a way that was not indicated on the device application (e.g., used for joint injection without a bone graft, if the initial product application involved mixing with bone graft).

### Consensus by stakeholders

Logistics of SOP implementation, primarily to comply with product labeling and documentation on the EMR, necessitated collaboration with operations technology teams. Integration of required regulatory elements into procedure document templates, for example, appeared differently for each clinic based on unique PRP uses (e.g., joint injection versus topical wound application) and kits. Consent documentation was verified but not required to be uniform amongst clinic sites.

## Discussion

Standardization of PRP best practices and compliance with regulatory standards are needed to minimize variability, promote safety, and operationalize as the scale of use grows. Core IP categories of risk identified during an internal audit and outlined for broader use here provide a framework for IP teams, HCP, and clinical groups utilizing PRP to investigate the scope of practice and opportunities to standardize approaches. This framework may be adapted to several settings according to clinic type and PRP treatment goals. Importantly, protocols for PRP reflect blood product regulatory standards which are traditionally conceptualized by HCP as applying to blood transfusion, which may add to confusion and reinforces the need to raise awareness and standardize practices.

Our experience highlights the extent of use was much wider than initially understood, after PRP was identified in a single practice setting. While PRP at this institution centered around orthopedics, a vast array of other subspecialty uses exist. Within orthopedics, PRP is a type of orthobiologic treatment given as a joint or tendon injection for musculoskeletal injury or arthritis.^16–18^ Skin treatments include alopecia, facial rejuvenation (e.g., facelifts, microneedling including for acne scars) and wound healing by topical application.^19,20^ PRP is utilized adjunctively in dental and oral surgery procedures to promote tissue regeneration and bone socket preservation, such as following tooth extraction or implants.^21^ Maxillofacial applications may uniquely include a tissue component with platelet-rich fibrin or mixing PRP with auto- or allograft bone.^22^ PRP use for wound and bone infections, while largely based on *in vivo* and observational data, is also emerging.^23^

To gain clarity about the number and type of PRP procedures performed at this institution, clinic locations and set ups, HCP involved, kits used, centrifuge models, consent forms, labeling and documentation practices, the IP team reached to core clinic stakeholders, vendors, supply chain personnel, and completed multiple on-site clinic visits. Additionally, while the total number of PRP procedures may have been underestimated based on incomplete capture by CPT codes that did not include PRP procedures received via a bundled care service, CPT codes were nevertheless valuable to approximate the PRP scope. This should be considered by other institutions if relying on CPT codes to quantify PRP procedures.

Variability in practice not only stems from distinct anatomic applications of PRP but also is impacted by different resources available to HCP and healthcare settings. Even within the same healthcare system, between clinics and operating rooms, there may be separate set ups for PRP that affect workflow and feasibility of adhering to requirements by regulatory agencies including AABB, OSHA, and FDA. For example, certain centrifuge models do not require manual transfer of the blood product into a new tube between spins, which reduces the risk of contamination or injection safety adverse event. However, these models may not achieve sufficient platelet concentrations of the final product and therefore are possibly less desirable for orthopedic applications. Spatial challenges in clinics may require the centrifuge to be located outside of the clinical area where PRP is performed, reinforcing the need for reliable application of patient identifiers and product labeling to avoid mix-ups between patients.

Perception of risk by HCP performing PRP may be minimized by the autologous nature, in contrast to protocols and regulations concerning other blood products derived from different donor(s) and infused intravenously. This rationalization could lead to more relaxed or limited IP practices. While the PRP process is not subject to certain AABB requirements for transfusion of other blood products, there remain numerous overlapping infection and safety risks which support the need to standardize PRP.

PRP process standardization aims to avert risks of microbial contamination at any step and BBP exposure. The latter has been described with nonsterile injection practices, including likely HIV transmission in spas performing microneedling facials in a CDC Morbidity and Mortality Weekly Report.^24^ Post-procedural infectious complications of PRP are not well described in current literature, suggesting that adoption of surveillance strategies would be valuable.^25^ Adjacent to PRP, outbreaks of bacterial septic arthritis associated with intra-articular steroid injections have been tied to breaches in hand hygiene, unsafe injection practices, and medication preparation too far in advance of the procedure.^26^

Integrating reporting requirements of PRP as a blood product into workflows that are not typically associated with this, such as joint injection with steroid or topical wound and skin treatments, also requires expertise from technology teams to streamline documentation for providers. Capturing necessary reporting elements remains a priority as PRP use and scope expands across both ambulatory and inpatient landscapes. Medical-legal vulnerabilities have also been raised regarding lack of standardization around informed consent documentation specific to PRP.^27,28^ Healthcare systems should ensure that consent forms in conjunction with patient educational materials are tailored to the procedure type (e.g., orthopedic versus dermatologic), communicate essential process steps, potential risks, and strategies to mitigate adverse events.

Limitations of this assessment include a single-center experience with PRP focused on orthopedic use. Standardized guidance implementation will assume various forms predicated on PRP uses, clinic resources including staffing and feasibility to integrate process flows. Regulatory gaps remain for a process that is not yet formally FDA approved, though this guidance offers a useful tool to raise awareness of PRP and suggests further standardization would be beneficial to unify safety practices more broadly.

As more data becomes available on the efficacy of PRP as a therapeutic intervention for various conditions, particularly musculoskeletal injury and arthritis, an increase in use may be expected. Establishment of best practices is needed to mitigate risks of infection, ensure safe handling of PRP by HCP, and operationalize required reporting elements into provider workflows and healthcare information technology systems.

## Supporting information

Stern et al. supplementary material Stern et al. supplementary material

## Supplementary material

To view supplementary material for this article, please visit https://doi.org/10.1017/ice.2025.10316
