# Platelet-rich plasma therapy: key infection prevention practices and strategies for safety risk reduction – CORRIGENDUM

**DOI:** 10.1017/ice.2026.10457

**Published:** 2026-06

**Authors:** Rebecca A. Stern, Jennifer Andrews, Katherine Bashaw, Thomas R. Talbot

The author wishes to clarify certain lines in the above article.

Lines 50-52 have been corrected to state: “The nuances of managing a blood product are prudent to incorporate into risk evaluation and logistics, though PRP is technically not classified as a blood product per the Association for the Advancement of Blood & Biotherapies (AABB).”

Lines 199-203, the sentence “As a blood product, PRP regulatory oversight falls under the United States Food and Drug Administration (FDA) Center for Biologics Evaluation and Research (CBER), though is exempt from the 21 Code of Federal Regulations 1271.15(b) standard as an autologous product that is readministered to the patient during the same procedure” has been replaced with the following statement: “Importantly, PRP is not treated as a blood product per AABB, though we used standards around blood product administration as best practice for PRP preparation and administration.”

The clarifications have been made and the online HTML and PDF versions updated.
