# Supplementary material for: Platelet-rich plasma therapy: key infection prevention practices and strategies for safety risk reduction
Source: Infect Control Hosp Epidemiol. 2025 Oct 16;47(1):1–5. doi: 10.1017/ice.2025.10316 (PMC12780834; doi:10.1017/ice.2025.10316)
Supplement: Stern et al. supplementary material [file S0899823X25103164sup001.docx]

**Supplement Table 1. PRP Audit Tool for Infection Prevention Best Practices**

| **Equipment and PRP Procedure Kits** | **Yes / No** |
| --- | --- |
| Kits are FDA-cleared |  |
| Centrifuge machines are cleaned after each point of care use per MIFU |  |
| Centrifuge is located in the same room where PRP preparation and administration are performed, whenever possible, or appropriate labeling and transportation of biohazard practices are employed |  |
| Preventive maintenance is performed at least annually on centrifuge |  |
| **Before the Procedure** |  |
| Ensure no contraindications to receive PRP |  |
| Patient education is completed |  |
| Consent is completed (verbal consent by provider observed by another staff member, and/or written consent signed and upload into EMR) |  |
| Time out is completed immediately before the procedure (observed by secondary staff member and/or in real-time documented in EMR) |  |
| Anticoagulant, if indicated, is drawn up per MIFU |  |
| **Procedure Set Up** |  |
| Sterile field is set up as close to the procedure time as possible |  |
| If sterile field is prepared in advance, it is covered with a sterile drape and stored away from high traffic areas to prevent contamination |  |
| Medication preparation is done no more than 4 hours before use |  |
| Medications are labeled with medication name, dose, preparation time |  |
| Verify sterile integrity of PRP kit (ensure package is intact, not expired) |  |
| Patient identification labels are added to every single-use container, syringe, or other kit device being used |  |
| Patient label includes the following: patient name, MRN, date of product expiration, date/time product collected, name of HCP who collected specimen |  |
| Document kit lot number and expiration date in EMR |  |
| **PRP Preparation** |  |
| MIFU followed for the kit being used |  |
| Phlebotomy is completed (blood volume per MIFU for designated treatment). If ultrasound guidance is used, a sterile probe cover is recommended. |  |
| Appropriate PPE is worn to prevent any splash |  |
| If transfer of blood and/or PRP product is required to another room (i.e., centrifuge in separate room from phlebotomy or treatment), it is transported in a leakproof biohazard bag and labeled appropriately |  |
| Clean the valves of the PRP separator devices / tubes with alcohol before accessing the port |  |
| Centrifugation is completed per MIFU |  |
| Final PRP product is labeled appropriately: patient name, MRN, PRP expiration date/time, autologous blood product designation, HCP name who prepared the product |  |
| **PRP Administration** |  |
| Patient identifiers are verified by two HCP on labeled final PRP product |  |
| Performed within 4 hours of PRP preparation (or per MIFU/product specifications) |  |
| Skin antisepsis is performed (e.g., chlorhexidine, or per protocol) |  |
| **Post-PRP Administration** |  |
| Waste is disposed of in appropriate containers (i.e. sharps, biohazard) |  |
| Centrifuge and counterbalances are cleaned in accordance with MIFU |  |
| Environmental cleaning is completed for workstations, patient equipment and care areas |  |

Assess throughout for hand hygiene, aseptic technique, standard precautions, and injection safety.

Abbreviations: MIFU, Manufacturer’s Instructions for Use; EMR, Electronic Medical Record; MRN, medical record number; HCP, healthcare personnel; PPE, personal protective equipment.
